# Floral organ MADS-box genes in *Cercidiphyllum japonicum* (Cercidiphyllaceae): Implications for systematic evolution and bracts definition

**DOI:** 10.1371/journal.pone.0178382

**Published:** 2017-05-31

**Authors:** Yupei Jin, Yubing Wang, Dechun Zhang, Xiangling Shen, Wen Liu, Faju Chen

**Affiliations:** Biotechnology Research Center, China Three Gorges University, Yichang, Hubei Province, P. R. China; Hainan University, CHINA

## Abstract

The dioecious relic *Cercidiphyllum japonicum* is one of two species of the sole genus *Cercidiphyllum*, with a tight inflorescence lacking an apparent perianth structure. In addition, its systematic place has been much debated and, so far researches have mainly focused on its morphology and chloroplast genes. In our investigation, we identified 10 floral organ identity genes, including four A-class, three B-class, two C-class and one D-class. Phylogenetic analyses showed that all ten genes are grouped with *Saxifragales* plants, which confirmed the phylogenetic place of *C*. *japonicum*. Expression patterns of those genes were examined by quantitative reverse transcriptase PCR, with some variations that did not completely coincide with the ABCDE model, suggesting some subfunctionalization. As well, our research supported the idea that thebract actually is perianth according to our morphological and molecular analyses in *Cercidiphyllum japonicum*.

## Introduction

*Cercidiphyllum japonicum* Sieb. Et Zucc. is a tertiary relic plant and only occurs as a species of east Asian flora. Paleontology research shows that it was once widely distributed in the northern hemisphere. Due to quaternary glaciations, it is now only sporadically found in China and Japan [1,2]. As a cretaceous relic, *C*. *japonicum* has considerable presence as a tree with colorful leaves. The tree displays typically colored leaves showing amaranthine in the spring, emerald in the summer, golden in the fall and carmine in the winter. As well, it has great economic value given that its fruits and leaves can be used as medicines and the bark is used for tannic extracts. Furthermore, its dioecious, achlamydeous and extreme simplification inflorescence makes it an ideal material for the study of sexual differentiation and regulation of floral development.

Since it was established by Siebold and Zuccarini in 1846 [3], the systematic position of *C*. *japonicum* has always been in dispute. In the early years, researchers classified it according to its morphology and it was once placed in the *Magnoliaceae* [4]. Baillon [5] proposed that *Cercidiphyllum* may be closely related with *Hamamelidaceae* plants, which wasapproved later and *Cercidiphyllum* was taken into the *Hamamelidaceae* [6]. On the other hand, Van Tieghem put forward that *Cercidiphyllum* should be its own family, a proposal generally accepted [7]. Much later, *Cercidiphyllum* was placed in *Trochodendrales* [8], *Hamamelidales* [9] or *Cercidiphyllales* [10] and *Cercidiphyllaceae* was regarded as the bond connecting *Hamamelidaceae*, *Trochodendrales* and *Magnoliales*. With sequence analysis, the molecular phylogeny of *rbc*L showed that *Cercidiphyllaceae* is close to *Daphniphyllaceae*, *Hamamelidaceae* and *Saxifragaeeae*. Combining their morphological characteristics, both *Cercidiphyllaceae* and *Daphniphyllaceae* should be classified with *Hamamelidales* [11]. Analysis of matK sequences declared that *Cercidiphyllaceae* has a distant relationship with *Tetracentracea* [12]. The APG II [13] and APG III [14] classification systems put *Cercidiphyllum* as an independent family in *Saxifragales*. Combining the floral morphogenesis, the type of vascular perforated plate and anatomical characteristics of *Cercidiphyllaceae*, Yan et al. [15] considered it was suitable to place *Cercidiphyllum* into *Saxifragales*. But the floral morphology and developmental processes were quite distinct from other *Saxifragales* plants. Since flowers are the most conserved organs for angiosperms, it is of great importance to investigate the systematic process according to the floral identity genes.

The ABCDE-model is the most acceptable model explaining flora development. In this model, A- and E-class genes determine sepal formation. A-, B- and E-class genes are responsible for petals. Stamens are determined by B-, C- and E-class genes, while C- and E-class genes determine the identity of carpels. D-class genes are involved in ovule development [16–18]. Almost all genes execute A, B, C, D and E functions, *APETALA1(AP1)*, *PISTILLATA(PI)* and *APETALA3(AP3)*, *AGOUMOUS(AG)*, *AGOUMOUS-Like11(AGL11)* and *SEPALLATA(SEP)* lineages belong to the MIKC-type MADS-box family, except for *APETALA2(AP2)*. Studies showed that these genes have the similar structure consisting of M, I, K and C domains with high conservation. B-/C-class genes were relatively conserved in function of controlling pistilate and staminal development [19]. A-class genes were diversified; for example, *AP1* mutation resulted in the absence of petals in *Arabidopsis*, but a recent study, about the spiral flowers of *Nigella damascena*, claimed that the *AGL6*-lineage, rather than the *AP1*-lineage, is an A-class gene, which is the key regulator of sepal and petal development [20]. Modified models have been discussed in many species for clarifying special flower structures.

The flowers of *C*. *japonicum* were considered to be very special and hence some arguments were cropped up over its flora structures. Solereder [6] and Harms [21] believed that its outward ventral suture characteristics showed that the flowers were inflorescence, thinking that the orientation may be resulted from the absence of an opposite carpel. However, Swamy and Bailey [22] tried to draw arguments for the loss of a second carpel. Both Van Heel [23] and Endress [24] observed early developmental stages of *C*. *japonicum*. Their descriptions suggested that the flowers develop in a decussate way and that the bracts outside the first couple were not opposite while the second couple were. Moreover, they agreed that the perianth and nectar of *C*. *japonicum* were missing. Yan et al. [15] observed the morphogenesis of *C*. *japonicum* and concluded that the bracts were lanceolate, membranous, not phyllome and associated with carpel development and hence the so-called bracts should be tepals. By this token, the floral structure of *C*. *japonicum* still remains a controversial issue.

In other words, *C*. *japonicum* is the ideal material to investigate its sex differentiation and floral developmental mechanism. Our research based on the ABCDE model further confirms the systematic evolution of *C*. *japonicum* by analyzing MADS-box homologs. We discuss its floral structure on the basis of morphologic observations and relative genes expression patterns.

## Material and methods

### Plant materials

Flower buds were collected from *C*. *japonicum* growing under natural conditions in Beijing with the cooperation of Dr. Guoke Chen from Institute of Botany, the Chinese Academy of Sciences. One part of the buds were immersed in glutaraldehyde. The others buds for cloning were separated into seven parts-outer scale (OS), middle scale (MS), inner scale (IS), stamens (ST) or carpels (CA), juvenile leaves (LE), stipule (STI) and bracts (BR) and immediately frozen in liquid nitrogen and stored at -80°C until used.

### Isolation and identification of genes

Total RNA was extracted from floral buds using the EASYspin plant RNA Extraction Kit (Aidlab, China) following instructions from the manufacturer. First-strand cDNA was synthesized from 1 μg of the DNase I-treated RNA, using adaptor primers and M-MLV Reverse Transcriptase (TaKaRa, Japan). Initial amplification for core sequences were based on homologous cloning. The PCR reagents were composed of 1 μL cDNA, 0.5 μL of each primer (10 mM each), 2.5 μL Ex Taq buffer, 2 μL dNTP (2.5 mM each), 0.3 μL Ex Taq plymerase (TaKaRa, Japan) and adjusted with water to a final volume of 25 μL. PCR was performed with a 3 min 95°C denaturation step, followed by 35 cycles of 30 s at 95°C, 30 s annealing at 52–57°C, a 30–60 s extension at 72°C and a final extension period of 10 min. The PCR products were purified with the gel extraction kit (TaKaRa) and cloned into pMD18^®^-T vector (TaKaRa). Ligation products were transformed into Escherichia coli Top10 cells (Aidlab China) following instructions by the manufacturer. Then we used 3’ RACE and 5’ RACE system kits (TaKaRa) to obtain the 3’- and 5’-end sequences of each gene. Full-length cDNA of each gene was obtained by PCR-based cloning with gene-specific forward and reverse primers designed according to the corresponding 3’- and 5’-end sequences. Names and sequences of the primers used in this study are presented in Tables 1 and 2.

**Table 1:** A list of all primers used for gene cloning and qRT-PCR in this study.

name	core sequences		3'RACE		5'RACE		qRT-PCR	
primer	F	R	first	second	first	second	F	R
gene								
*CejaAP1*	AP1-F	AP1-R	AP1-F	3’AP1-2	qAP1-R	AP1-R	q-AP1-F	q-AP1-R
*CejaFUL*	FUL-F	FUL-R	FUL-F	3’AF	–		q-FUL-F	q-FUL-R
*CejaFUL-like*	–		3’AF	3’FULlike-2	q-FUL-like-R	5’FUL-like-2	q-FUL-like-F	q-FUL-like-R
*CejaAGL6*	AGL6-F	AGL6-R	AGL6-F	3’AGL6-2	–		q-AGL6-F	q-AGL6-R
*CejaAP3_1*	AP3-F	AP3-R	AP3-F	3’AP3-2	5’AP3-1	AP3-R	q-AP31-F	q-AP31-R
*CejaAP3_2*							q-AP32-F	q-AP32-R
*CejaPI*	PI-F	PI-R	3’PI-1	3’PI-2	q-PI-R	5’PI-2	q-PI-F	q-PI-R
*CejaAG1*	AG-F	AG-R	AG-F	3’AG1-2	q-AG1-R	5’AG1-2	q-AG1-F	q-AG1-R
*CejaAG2*				3’AG2-2	q-AG2-R	AG-R	q-AG2-F	q-AG2-R
*CejaAGL11*	AGL11-F	AGL11-R	AGL11-F	3'AGL11-2	q-AGL11-R	AGL11-R	q-AGL11-F	q-AGL11-R
*CejaActin*	actin-F	actin-R	–		–		q-actinF	q-actinR

**Table 2:** Sequence information of the primers listed in Table 1.

Primer	Primer sequences(5’ to 3’)	Primer	Primer sequences(5’ to 3’)
AP1-F	GAGGTTGCTTTGATTGTCTTCTC	5’FUL-like-2	AGAGAAGGAAGTGGTAGTGGTTGAG
AP1-R	TGAGGTCGAGCTCGTTCCTCCT	5’AP3-1	CCTACGCCTTGCTTGAGTAGCACC
FUL-F	GATCAATAGGCAAGTGACGTTTTC	5’PI-2	GGCTTTTATCCTCCTCCGCCAACAT
FUL-R	CATAAGTAGGTTCTTTCTTGACC	5’AG1-2	TTCCGAGAGTCGAATGGCGGAGA
AGL6-F	GAGAGAGAATGGGGAGAGGAAG	q-actinF	AAGATCTGGCATCACACTTTCTACA
AGL6-R	CGGAGGTCTTCCATTTGTTCT	q-actinR	ATAAATTGGAACTGTATGGCTCACC
AP3-F	GGTCTCTTCAAGAAGGCAAATG	q-AP1-F	GCATCATCCTTCCTATTACCACA
AP3-R	CTTGCAAGTTTCAATCTGATTAGTG	q-AP1-R	AAATCATAAATTCATAACCAGCT
PI-F	ATGGGGAGAGGGAAGATTGAGAT	q-FUL-F	ACCAGACAGGAAGTAGTGGAGGA
PI-R	GTAAATTTGGCTGGATTGGCTGCAC	q-FUL-R	ATGCCAGAGCAATTAATATAGGA
AG-F	CAAGTCACCTTCTGTAAGCG	q-FUL-like-F	CTCAACCACTACCACTTCCTTCTCT
AG-R	CTCATTTTCAGCTATCTTTGCTCG	q-FUL-like-R	GGTGGTTGGAAAGAGTTTCCATCCT
AGL11-F	GATGCTGAAGTTGCCCTCAT	q-AGL6-F	CATCCCTCTCAATCCAACCCCAT
AGL11-R	CCATGTCTGCTTGCTGAAGCCTCTC	q-AGL6-R	GATTATTAAAGGACCCATCCCTGGA
actin-F	AAGATCTGGCATCACACTTTCTACA	q-AP31-F	ATTAGGCAGAGGATGGGTCAGAA
actin-R	GACCGGACTCATCATACTCT	q-AP31-R	AGGAGACCTCTGTGTATTTGTTC
3’AP1-2	AGCATGGAGAAAATCCTTGAACG	q-AP32-F	ATGAGATTAGTATCGCGGGATCAC
3’AF	GAGGTCGGGTTGATCGTCTTCTCCAC	q-AP32-R	CCTCCATTTTGATATCCAAGAACAG
3’AF	TGAAGTCTTGCAAAGGAACCTAAGG	q-PI-F	GGCTATGGAAGATAATGTGAGGC
3’AGL6-2	GCTTTCTGTGCTGTGTGATGCTG	q-PI-R	CCTCTATTACAAACCCGACAAAGCA
3’AP3-2	GAGGTTAATAACAAACTGCGGC	q-AG1-F	TCTCCGCCATTCGACTCTCGGAA
3’PI-1	GAGAACTCAACTAACAGGCATGTGA	q-AG1-R	GGTTCCCTCCACAGAAGGTAAAC
3’PI-2	TGGGAAGAAGTTGTGGGATGCTAAG	q-AG2-F	TGATGACAGTACCTGTGTACGAGGC
3’AG1-2	CAAAGTGCGCAAATCGTGAGTTTG	q-AG2-R	GAAGGGCAGGGATAGAACTCCAGAT
3’AG2-2	GGAAACAAATCCAGGATACACAAAG	q-AGL11-F	CAAGATAGCAGAATCCGAGAGGC
3'AGL11-2	CTGGAGAATAGACTTGACCGAGG	q-AGL11-R	ATGCAGAGATCCATAACAGTGGC

### Sequence alignments and phylogenetic analysis

Selected sequences were downloaded from the National Center for Biotechnology Information GenBank. The taxa were selected on the basis of aligning results and the representative angiosperm classification according to the APGIII system (APGIII, 2009). Only one taxon provided relatively complete cds and was chosen per order. Alignments were conducted by Clustal X 2.0 using protein sequences and phylogenetic trees were formed by software MEGA7.0 using the Neighbor-Joining (NJ) and Maximum Likelihood (ML) Method. *Gnetum gnemon* and *Picea abies* were chosen as outgroups. Relative species and accession numbers are shown in Table 3. Support for the branches was assessed using bootstrap analysis with 1000 replicates.

**Table 3:** All the MADS-box proteins in protein sequence comparisons and phylogenetic analysis.

Protein	Species	Accession number
CejaAP1	*Cercidiphyllum japonicum* (this paper)	KY285019
CejaFUL	*Cercidiphyllum japonicum* (this paper)	KY285024
CejaFUL-like	*Cercidiphyllum japonicum* (this paper)	KY285022
DAL1	*Picea abies*	CAA56864
GGM1	*Gnetum gnemon*	CAB44447
FL2	*Dicentra eximia*	AGX01574
MpMADS15	*Magnolia praecocissima*	BAB70749
CsAP1	*Chloranthus spicatus*	AAQ83693
BUseFL2	*Buxus sempervirens*	ABG49514
PAteFL1	*Pachysandra terminalis*	ABG49521
PAteFL2	*Pachysandra terminalis*	AAP83389
TraFUL1	*Trochodendron aralioides*	ABQ85944
TraFUL2	*Trochodendron aralioides*	ABQ85945
FL1	*Dicentra eximia*	AGX01534
GumaFUL-like	*Gunnera manicata*	AFO68793
VFUL-L	*Vitis vinifera*	NP_001268211 XP_002281526 XP_002281532
VFUL	*Vitis vinifera*	ACZ26529
HeaFL	*Heuchera americana*	AAP83373
AcFUL-like	*Actinidia chinensis*	ADU15471
RhFUL	*Rosa hybrid cultivar*	ACS74808
MADS4	*Betula pendula*	CAA67968
HeaFUL	*Heuchera americana*	AAP83374
CsFUL	*Corylopsis sinensis*	AAP83371
GlmAP1a	*Glycine max*	ABZ80361
FUL	*Arabidopsis thaliana*	OAO94650
LcAP1	*Litchi chinensis*	AEY55406
PpAP1-2	*Pyrus pyrifolia*	AJW29022
MADS5	*Betula pendula*	CAA67969
CcAP1	*Carya cathayensis*	AHI85952
CoarFUL	*Coffea arabica*	AHW58040
AcFUL	*Actinidia chinensis*	ADU15472
SpFUL	*Spinacia oleracea*	ACE75945
SpAP1-1	*Spinacia oleracea*	ACE75943
GsAP1	*Gentiana scabra*	BAS0447
CoarAP1	*Coffea arabica*	AHW58038
SiAP1	*Sesamum indicum*	AIS82596
CokoAP1	*Cornus kousa*	AGA61753
PalaAP1	*Paeonia lactiflora*	AGH61290
VvAP1	*Vitis vinifera*	NP_001268210 XP_002263170
HeaAP1	*Heuchera americana*	AAP83372
CsAP1	*Corylopsis sinensis*	AAP83370
CasiAP1	*Camellia sinensis*	AIC75372
CpAP1	*Cyclamen persicum*	BAK09614
MnAP1	*Morus notabilis*	EXB44879
ZjAP1	*Ziziphus jujuba*	ACG70964
MADS3	*Betula pendula*	CAA67967
CcAP1	*Carya cathayensis*	AHI85952
CisiAP1	*Citrus sinensis*	AAR01228
AP1	*Arabidopsis thaliana*	CAA78909
VuAP1	*Vigna unguiculata*	BAJ22385
PpAP1-3	*Pyrus pyrifolia*	AJW29025
FaAP1	*Fragaria x ananassa*	AFA42327
PsAP1-1	*Populus simonii x Populus nigra*	AGR88912
PeAP1	*Passiflora edulis*	AER30447
CejaPI	*Cercidiphyllum japonicum* (this paper)	KY285023
CejaAP3_1	*Cercidiphyllum japonicum* (this paper)	KY285020
CejaAP3_2	*Cercidiphyllum japonicum* (this paper)	KY285021
PrDGL	*Pinus radiata*	AAF28863
GGM2	*Gnetum gnemon*	CAB44448
AmPI	*Amborella trichopoda*	BAD42443
Nyod.PI	*Nymphaea odorata*	ADD25210
NymPI	*Nymphaea sp*.	AAR87705
IlflPI	*Illicium floridanum*	AAY25570
MpMADS8	*Magnolia praecocissima*	BAB70743
PeamPI	*Persea americana*	AAR06672
EgGLO	*Elaeis guineensis*	XP_010911271
CsPI	*Chloranthus spicatus*	AAF73939
PjPI	*Phalaenopsis japonica*	AJG41730
TraPI1	*Trochodendron aralioides*	ABQ85946
TraPI2	*Trochodendron aralioides*	ABQ85947
PsPI	*Paeonia suffruticosa*	AEE98378
RbFPI1	*Ribes diacanthum*	AHY19022
DiiPI1	*Dillenia indica*	ABR68541
PrpsPI	*Prunus pseudocerasus*	AIU94284
PMADS2	*Jatropha curcas*	XP_012078322
PdPI	*Populus deltoides*	ABS71831
AcPI	*Actinidia chinensis*	ADU15475
GLO	*Camellia oleifera*	AJN00602
NymAP3	*Nymphaea sp*.	AAR87701
AmAP3_1	*Amborella trichopoda*	BAD42444
MaspAP3	*Magnolia sprengeri*	AFN68915
MAprAP3	*Magnolia praecocissima*	BAB70742
CsAP3	*Chloranthus spicatus*	AAR06664
PAteAP3_1	*Pachysandra terminalis*	ADC79700
RbMAP3	*Ribes diacanthum*	AHY19023
MCAP3	*Micranthes careyana*	ABF56142
CopAP3	*Corylopsis pauciflora*	ABF56128
TroAP3	*Trochodendron aralioides*	ABE11601
PaLaAP3_1	*Paeonia lactiflora*	AGH61291
MadMdTM6	*Malus domestica*	NP_001315678 XP_008344258
PTD	*Populus trichocarpa*	AAC13695
HmTM6	*Hydrangea macrophylla*	BAG68950
GtAP3_1	*Gunnera tinctoria*	AAR06687
GmAP3	*Gunnera manicata*	AFO68771
SxcTM6	*Saxifraga careyana*	ABF56143
DiiTM6	*Diilenia indica*	ABR68544
CejaAG1	*Cercidiphyllum japonicum* (this paper)	KY285015
CejaAG2	*Cercidiphyllum japonicum* (this paper)	KY285016
CejaAGL11	*Cercidiphyllum japonicum* (this paper)	KY285018
DAL2	*Picea abies*	CAA55867
GGM3	*Gnetum gnemon*	CAB44449
AmAG	*Amborella trichopoda*	AAY25577
MAwuAG	*Magnolia wufengensis*	AEO52692
MisiAG	*Magnolia sirindhorniae*	AGZ63865
LoAG	*Lilium hybrid cultivar*	AEK94071
EgAG1	*Elaeis guineensis*	AAW66881
AoAG	*Alpinia oblongifolia*	ABB92624
NuadAG	*Nuphar advena*	AAY25576
NymAG1	*Nymphaea sp*.	AAS45692
HtcAG	*Houttuynia cordata*	AAS45684
TraAG1	*Trochodendron aralioides*	ABQ85948
TraAG2	*Trochodendron aralioides*	ABQ85949
PasuAG	*Paeonia suffruticosa*	AGS12611
SxcAG1	*Saxifraga careyana*	AAS45705
VvAG	*Vitis vinifera*	NP_001268097 XP_002263066
JacuAG	*Jatropha curcas*	NP_001292936 XP_012091857
MAG	*Mangifera indica*	ACN97631
CmMADS2	*Castanea mollissima*	AAZ77747
KejaAG	*Kerria japonica*	AGZ01978
PMAG	*Prunus mume*	ABU41518
CoAG	*Cornus kousa*	AGA61751
CoarAG	*Coffea arabica*	AHW58037
SiAG	*Sesamum indicum*	AIS82595
DiiAG	*Dillenia indica*	ABR68545
PLENA	*Gunnera manicata*	AFO68768
LAG	*Liquidambar styraciflua*	AAD38119
Mople	*Misopates orontium*	CAJ44134
plena	*Antirrhinum majus*	BAI68391
GsAG1	*Gentiana scabra*	BAS04480
CoarPLE	*Coffea arabica*	AHW58047
GsAG2	*Gentiana scabra*	BAS04484
NyodAG3	*Nymphaea odorata*	ADD25206
SxcAG2	*Saxifraga careyana*	AAS45704
MADS10	*Malus domestica*	NP_001280931
PpAGL11_1	*Pyrus pyrifolia*	AJW29026
MADS5	*Vitis vinifera*	AAM21345
JacuAGL11	*Jatropha curcas*	XP_012073508
GrAGL11	*Gossypium raimondii*	XP_012447416
CisiAGL11	*Citrus sinensis*	XP_006478235
GmAGL11	*Glycine max*	NP_001236130
AGL11	*Arabidopsis thaliana*	AAC49080
LjAGL11	*Lotus japonicus*	AAX13306

### Gene expression analysis

For our semi-quantitative RT-PCR analysis, total RNA was extracted from seven parts described earlier. Each first-strand cDNA was synthesized using an oligo (dT)15 primer and the M-MLV reverse transcriptase kit. To precisely analyze the tissue-specific expression patterns of each lineage genes, real-time quantitative PCRs are conducted. The experiment was accomplished with SYBR premix Ex Taq (Takara, Japan) using the following program: 95°C for 30 s; 40 cycles of 95°C 5 s, and 60°C for 30 s. The beta-actin gene of *C*. *japonicum Cejaactin* is referred as internal reference.

### Morphological observations

Mature floral buds from pistillate and staminate flower of *C*. *japonicum* were dissected with a needle and photographed under a stereoscopic microscope. All parts were separately fixed overnight in glutaraldehyde (2.5% glutaraldehyde in a 25 mM sodium phosphate buffer, pH 6.8) at 4°C. After dehydration in a graded ethanol series, the specimens were introduced at a critical point into liquid CO_2_. The dried material was mounted and coated with gold-palladium using a Hitachi E-1010 sputter Coater. Specimens were examined using a FEI-Quanta 200F scanning electron microscope with an accelerating voltage of 15 kV.

## Results

### Morphological observations

**Fig 1:**
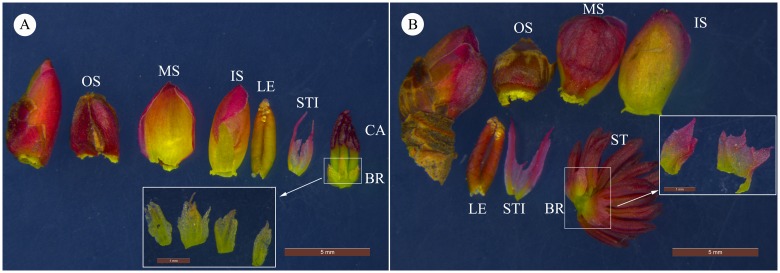
Morphology of *Cercidiphyllum japonicum* flowers. (A) Female inflorescence bud and dissections parts. (B) male inflorescence bud and dissections parts. OS = outer scale, MS = middle scale, IS = inner scale, ST = stamens, CA = carpels, LE = juvenile leaves, STI = stipule, and BR = bracts. Male and female inflorescence are showing the same outlook of OS, MS, IS, LE and STI.

The flowers of *C*. *japonicum* are small and inconspicuous, with similar flowering buds and leaf buds. The inflorescence has a juvenile leaf and a stipule which are embedded in three scales. The outer scales are russety, thick and sclerotic. The middle and inner scales are membranous, stretching out from the outer ones as they develop. When young, the middle and inner scales are peak green with a rose-red margin and turn yellowish with a red margin when mature. Juvenile leaves and stipules are found at the bottom of the pedicel. Juvenile leaves with transparent scrotiform glands in the margin are involute when they are wrapped in scales. The stipules are lanceolate, subtranslucent and membranous. The inflorescence of *C*. *japonicum* is highly simplified, with their pistillate inflorescence formed by four subtranslucent peak green bracts and 2–6 carpels, whose flat and upturned stigma is yellowish-green when young and turn scarlet when mature (Fig 1A). From our observations, we conclude that there are only two membranous bracts and several stamens whose heads are a bit sharp. The anthers are greenish when young and turn crimson when mature, with filaments almost did not elongate until when they are nearly mature (Fig 1B).

**Fig 2:**
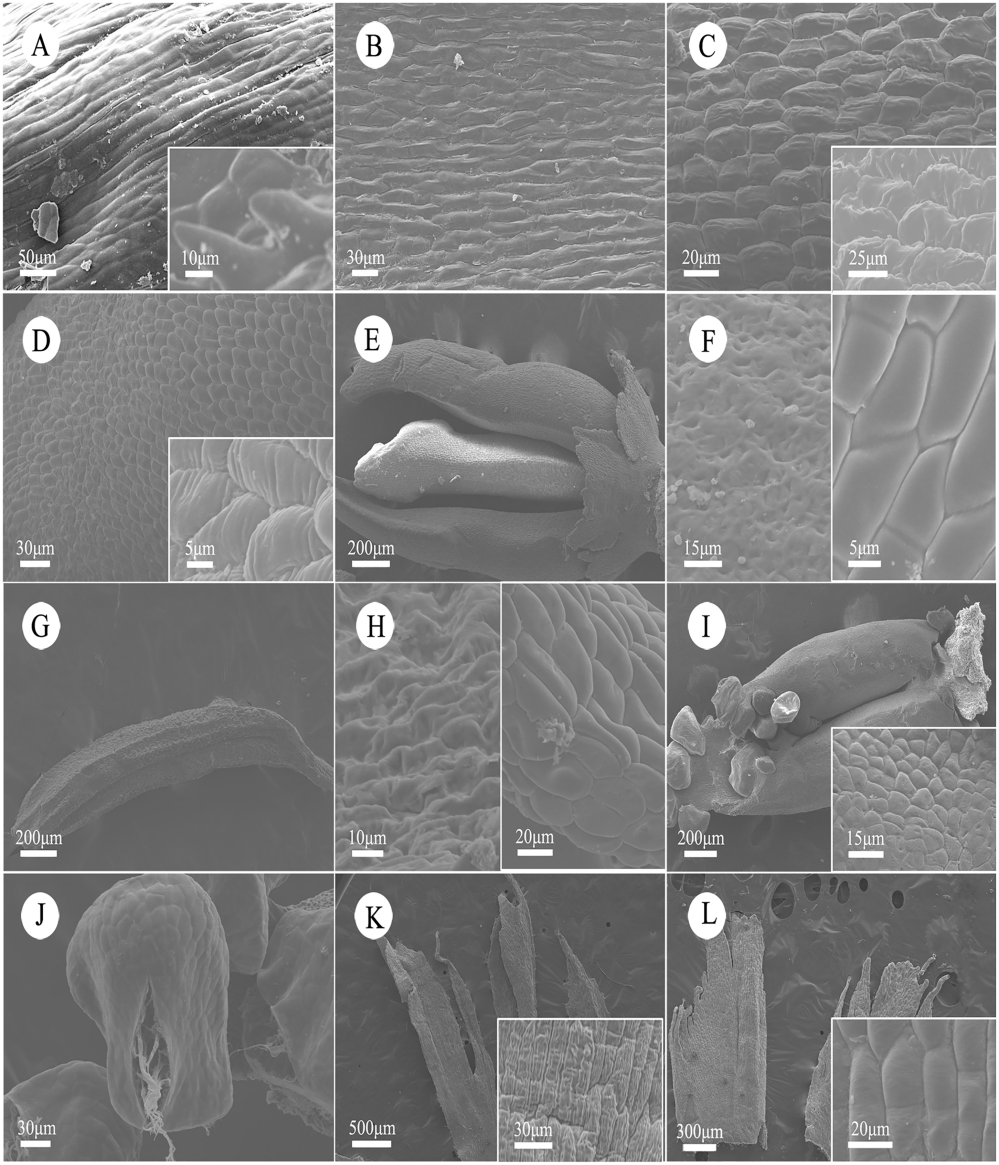
Epidermal cells of leaves and floral parts of *Cercidiphyllum japonicum*. Since male and female flowers are the same besides floral organs, so just female ones were displayed. (A) Abaxial (bar = 50 μm) and amplified (inset; bar = 10 μm) epidermal cells of a outer scale at mature stage. (B) Adaxial (bar = 30 μm) epidermal cells of a outer a scale at mature stage. (C) Abaxial (bar = 25 μm) and adaxial (inset; bar = 20 μm) epidermal cells of a inner scale at mature stage, showing irregular striation. (D) Abaxial (bar = 30 μm) and adaxial (inset; bar = 10 μm) epidermal cells of a inner scale at mature stage. (E) Carpels from a mature flower (bar = 200 μm). (F) Epidermal cells of a stigma (left; bar = 15 μm) and back (right; bar = 5 μm) of carpel. (G) A stamen from a mature flower (bar = 200 μm). (H) Surface of anther (left; bar = 10 μm) and filament (right; bar = 20 μm). (I) Juvenile leaves (bar = 200 μm) and the abaxial and amplified epidermal cells (inset; bar = 15 μm). (J) Epidermal cells of glands (bar = 30 μm). (K) Surface of a stipule (bar = 500 μm), showing relatively regular sculpturing (insert; bar = 30 μm). (L) Bracts (bar = 300 μm), showing middle slotted or tee or cross grooves (bar = 20 μm).

For an individual flower, the morphology of epidermal cells among the various parts-three scales, juvenile leave, stipule, stamen or carpel and bract-are clearly distinct. When comparing the male and female flowers, except for the carpels and stamens, the other corresponding parts of flowers do not show clear differences on epidermal cells. The abaxial epidermal cells on the outer scales are long, fibrous and relatively smooth except for a few short horns (Fig 2A). While the adaxial epidermis can be clearly distinguished, the cells are short, irregular and rough with a raised edge in the middle (Fig 2B). Most epidermal cells on both adaxial and abaxial sides of the middle scales are short and square, while cells on the edge are longer and with irregular prismatic protuberances (Fig 2C). The inside and outside epidermal cells on the inner scales are basically the same, regular and square in the middle, longer in the margin and straddle parallel grooves (Fig 2D). Epidermal cells on stigma are sunken and irregular in shape; it is hard to distinguish between individual cells. Cells on ventral sutures are square and arranged densely, while the peripheral cells are relative long and smooth (Fig 2F). The epidermal cells on the head of stamens and cells at the stomium of anther are spheroidal or square, but other places of the anthers are irregular, distorted strips, difficult to affirm as single cells (Fig 2G). Elsewhere, the filament cells are smooth and regular and elongated (Fig 2H). Cells of veins are larger and protuberant, while the mesophyll cells are smaller, round or square protuberances (Fig 2I). Epidermal cells of glands on the edge of juvenile leaves are nearly square and smooth (Fig 2J). The epidermal cells on the cusp of stipules are short and round and the margin consists of monolayer cells, while the lower cells are regular strip foundations with parallel contorted folds with spiny protuberances in the margin (Fig 2K). The epidermal cells on bracts are distinct ellipsoid with regular horizontal slender striate bulges and most of them are slotted in the middle or have tee or cross grooves (Fig 2L).

### Screenening and phylogenetic analysis of homeotic genes

Ten floral organ identity genes were obtained by homologous cloning and RACE methods. Among these, four clones were identical to *AP1*, *FUL*, *FUL-like* and *AGL6* genes. These genes were respectively referred as *CejaAP1*, *CejaFUL*, *CejaFUL-like* and *CejaAGL6*. Three B-class transcripts were identified and referred as *CejaPI*, *CejaAP3_1* and *CejaAP3_2*. Two C-classgene were called *CejaAG1*, *CejaAG2* and the only D-class homologous gene was named *CejaAGL11*. We performed phylogenetic analyses and constructed trees of each gene and classified them into four trees.

**Fig 3:**
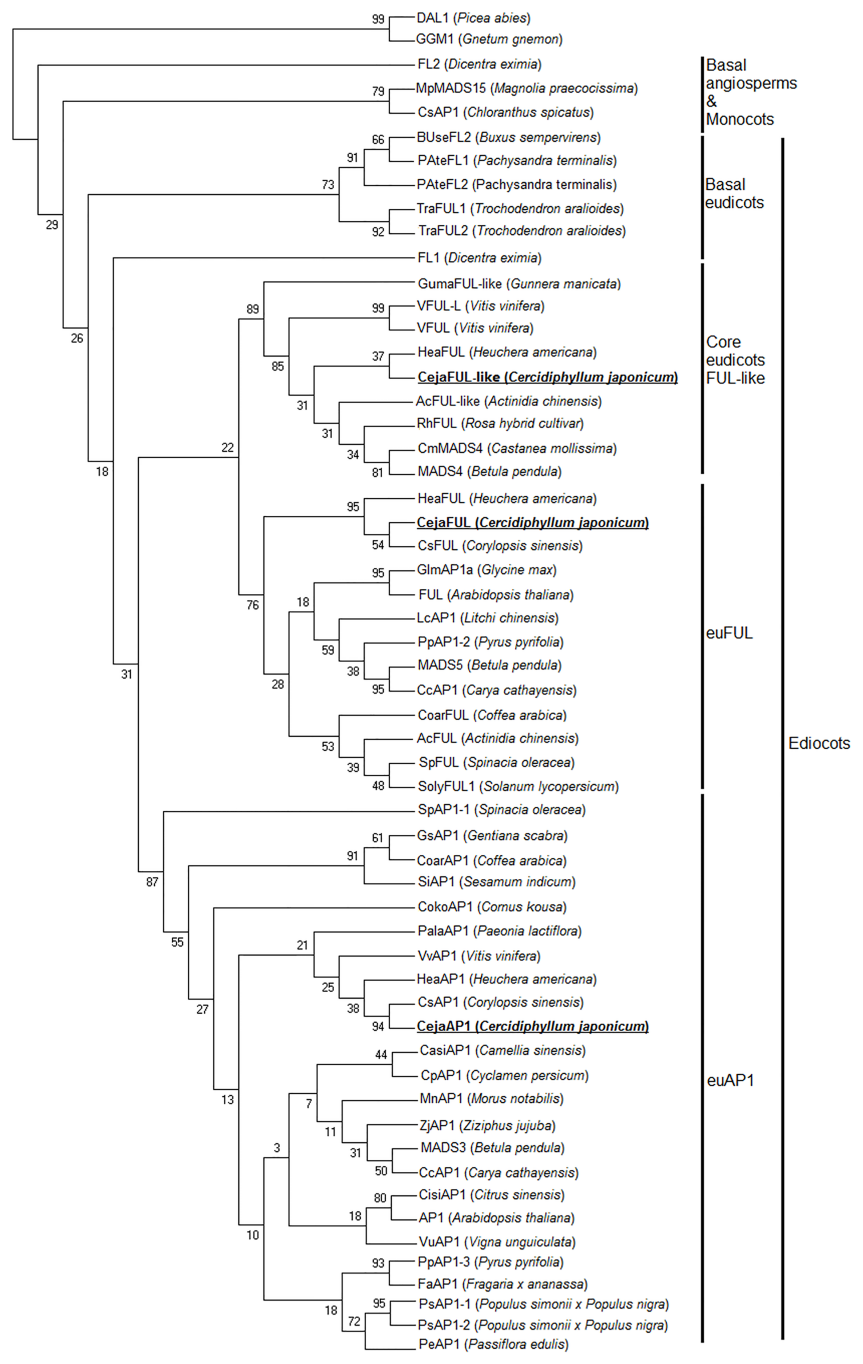
Phylogenetic analysis of A-class genes. A phylogenetic tree was built using the maximum-parsimony method through the program MEGA 7.0 based on the protein sequences of different species. *GGM1* and *DAL1* are used as outgroups. The percentage bootstrap values are indicated by numbers at the branch points.

**Fig 4:**
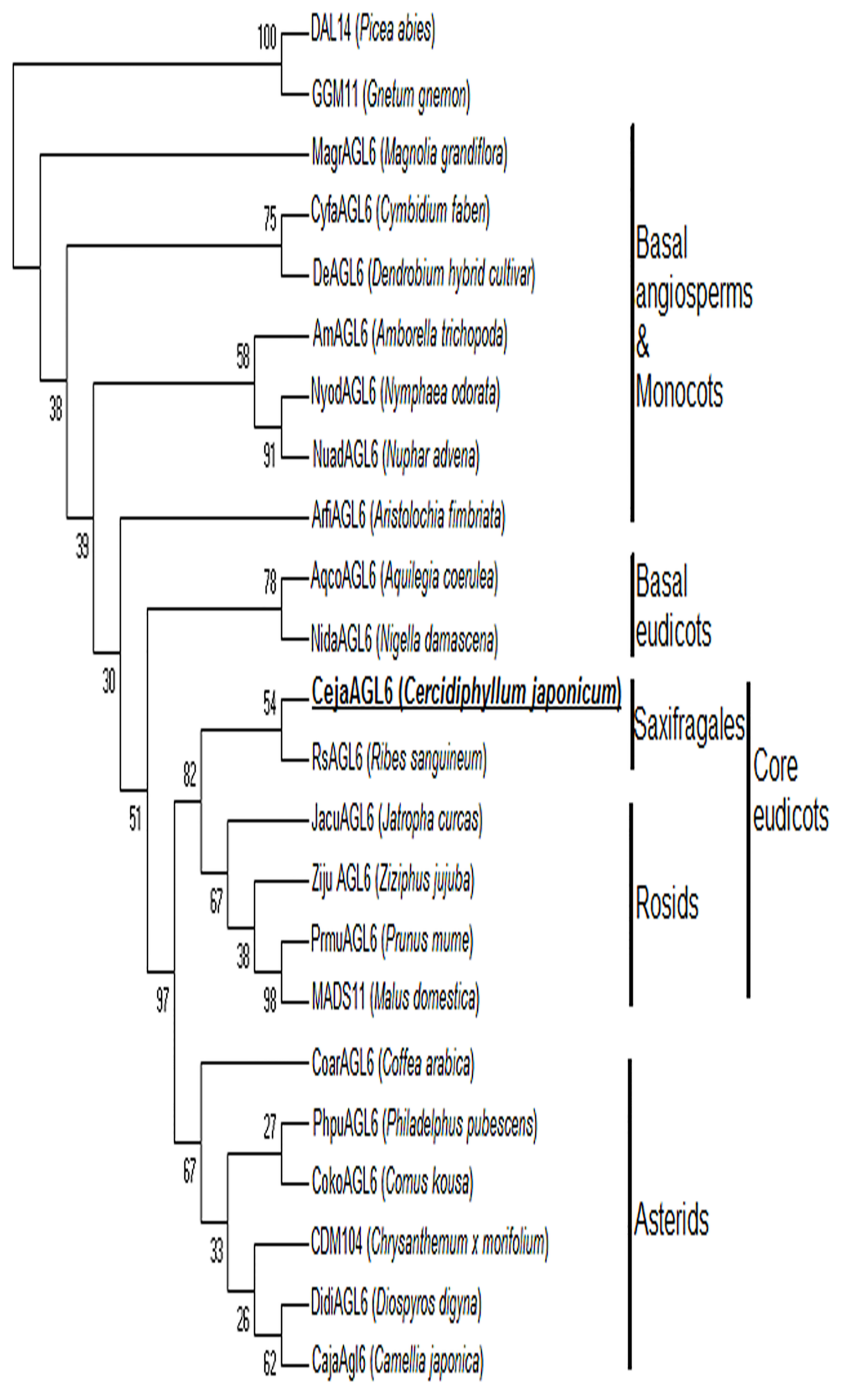
Phylogenetic analysis of *AGL6* lineages. A phylogenetic tree was built using the maximum-parsimony method through the program MEGA 7.0 based on the protein sequences of different species. *DGL14* and *GGM11* are used as outgroups. The percentage bootstrap values are indicated by numbers at the branch points.

According to the phylogenetic analysis of A-class genes, *CejaAP1*, *CejaFUL* and *CejaFUL-like* genes are respectively classified with *euAP1*, *euFUL* and *FUL-like* lineages in the basal core eudicots. *CejaAP1* and *CsAP1* of *Corylopsis sinensis* (Saxifragales) are sister groups, given bootstrap support under ML (94%) and form a clade with other eu*AP1* homologues of *Saxibragales*. *CejaFUL* and *CsFUL* of *Corylopsis sinensis* (Saxifragales) are sister groups and form a clade with *HeaFUL* of *Heuchera americana* (Saxifragales) with bootstrap support under ML (95%). *CejaFUL-like* also forms sister groups with *HeaFUL-like* of *Heuchera americana* (Saxifragales) (Fig 3). Since *AGL6* lineage was not a typical A-class gene, the phylogenetic tree of *CejaAGL6* was constructed only with its own lineage genes. The analysis shows that *CejaAGL6* groups with *RsAGL6* of *Ribes sanguineum* (Saxifragales) in the basal core eudicots (bootstrap 82%) (Fig 4).

**Fig 5:**
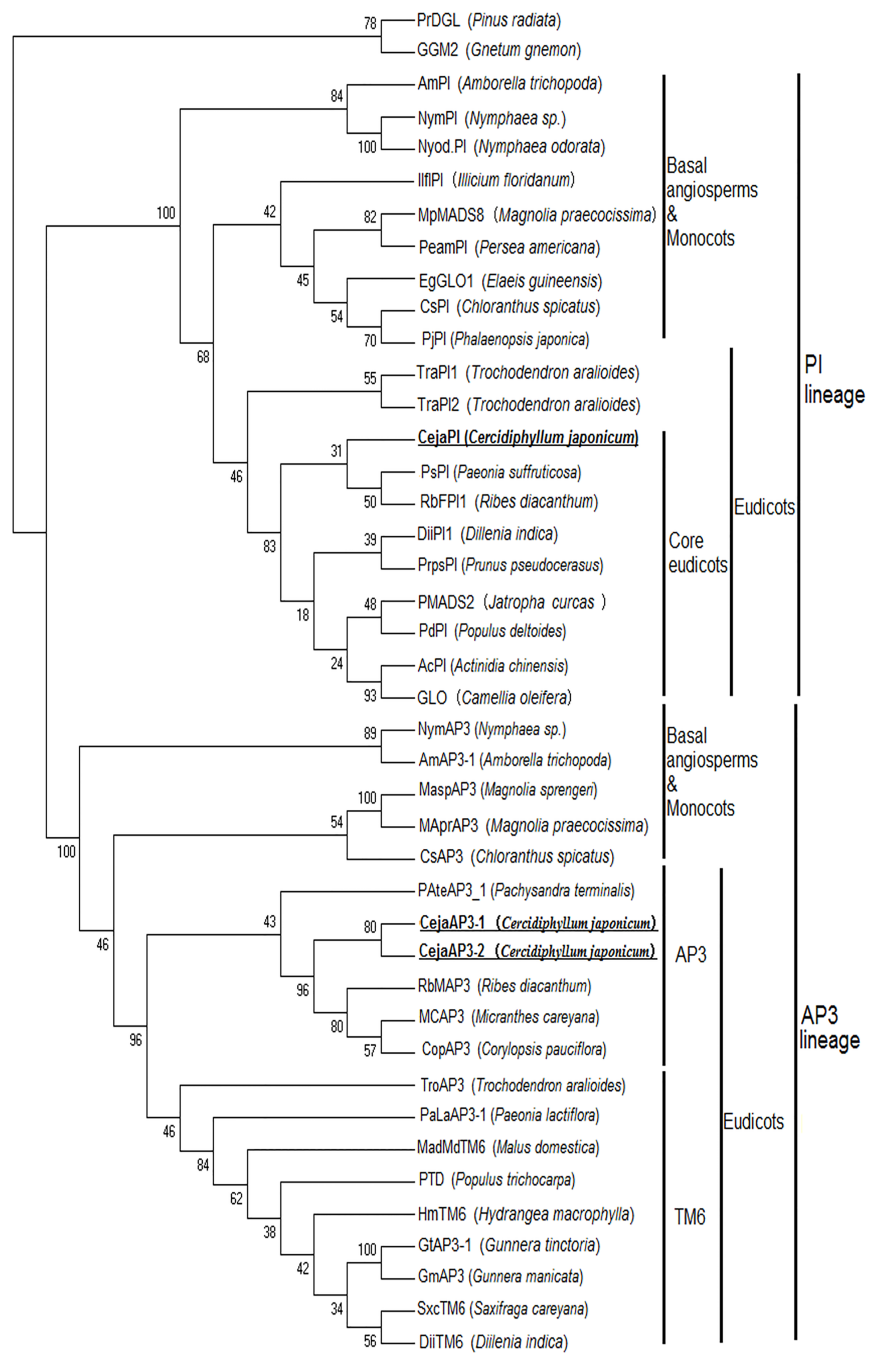
Phylogenetic analysis of B-class genes. A phylogenetic tree was built using the maximum-parsimony method through the program MEGA 7.0 based on the protein sequences of different species. PrDGL and GGM2 are used as outgroups. The percentage bootstrap values are indicated by numbers at the branch points.

*CejaPI*, the homologue of *PI in C*. *japonicum*, forms a sister group with *PsPI* of *Paeonia suffruticosa* (Saxifragales) and *RbFPI* of *Ribes diacanthum* (Saxifragales) even with low bootstrap support. *CejaAP3_1* and *CejaAP3_2* are grouped with *RbMAP3* of *Ribes diacanthum* (Saxifragales) and *CopAP3* of *Corylopsis pauciflora* (Saxifragales) with bootstrap support under ML (98%). This clade clearly branches off *TM6* lineages (Fig 5).

**Fig 6:**
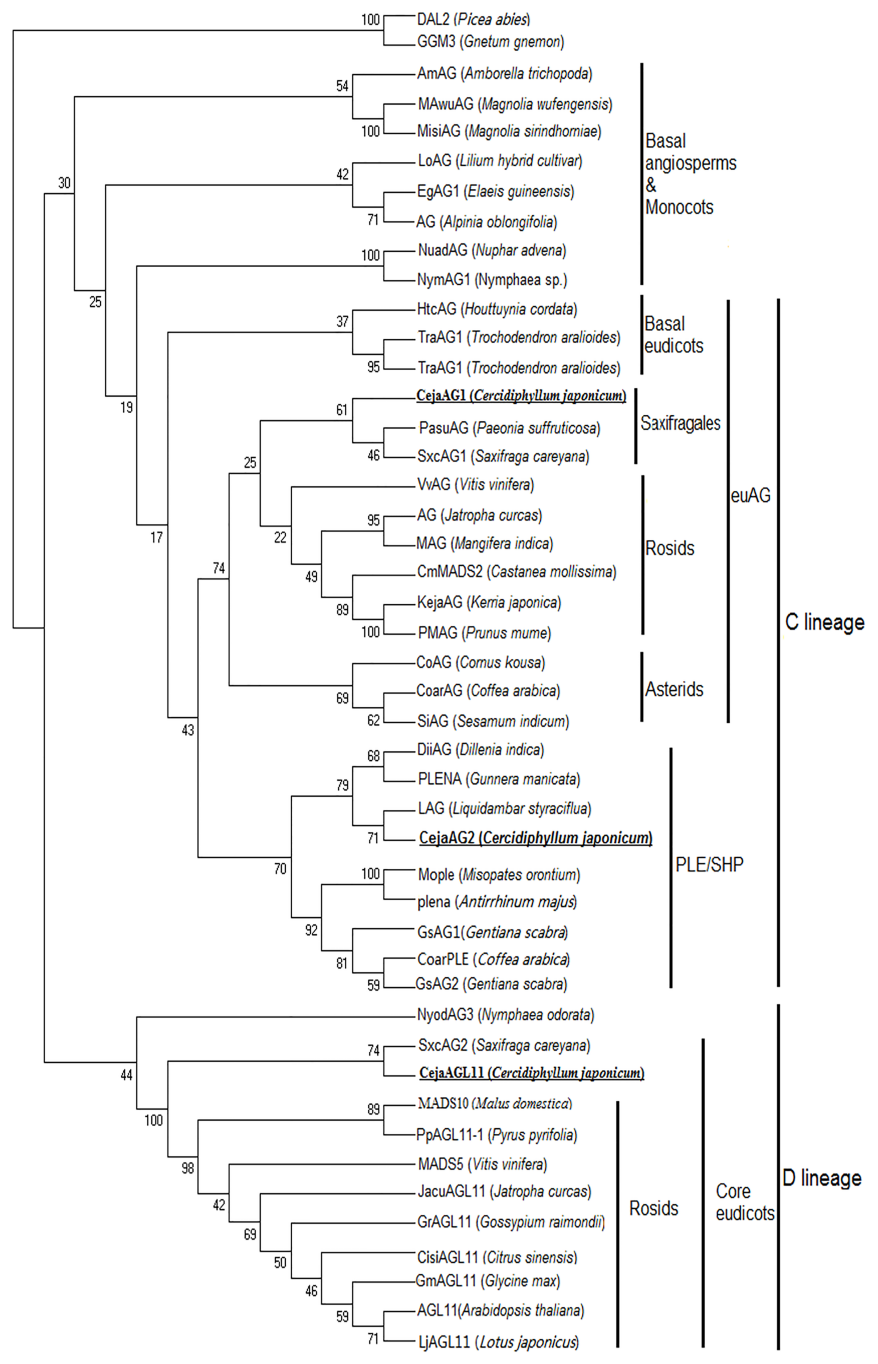
Phylogenetic analysis of C/D-class genes. A phylogenetic tree was built using the maximum-parsimony method through the program MEGA 7.0 based on the protein sequences of different species. *GGM3* and *DAL2* are used as outgroups. The percentage bootstrap values are indicated by numbers at the branch points.

Two C-class genes, *CejaAG1* and *CejaAG2*, were isolated; phylogenetic analysis showed that *CejaAG1* belongs to the *euAG* lineages and *CejaAG2* to the *PLE* lineages. *CejaAG1*, *PasuAG* of *Paeonia suffruticosa* (Saxifragales) and *SxcAG1* of *Saxifraga careyana* (Saxifragales) gather in a group with bootstrap support under ML (61%). *CejaAG2* and *LAG* of *Liquidambar styraciflua* (Saxifragales) is a sister group in the ML analysis (bootstrap 61% support). The only D-class gene *CejaAGL11* forms a clade with *SxcAG2* of *Saxifraga careyana* (Saxifragales) in the ML analysis (bootstrap 71% support) (Fig 6).

### Expression of ABCD Homologs in *C*. *japonicum*

**Fig 7:**
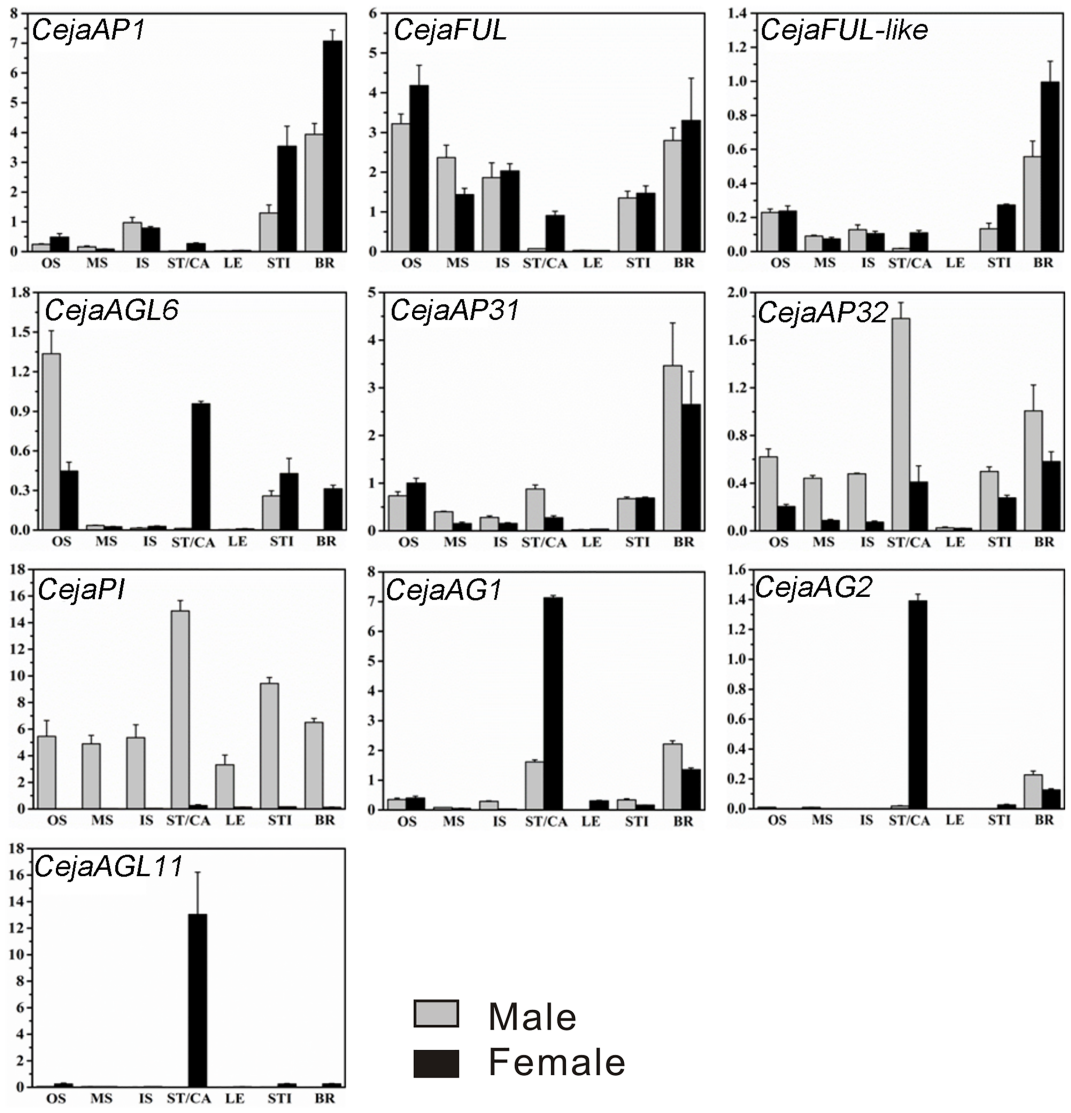
Expression patterns of floral organ identity genes. Real time qPCR was performed showing expression in different organs. The *CejaActin* was used as an internal reference. Values represent the means ± standard error of triplicates.

The expression patterns of the ABCD Homologs were analyzed by qRT-PCR. The expression patterns of these genes were shown in Fig 7. Except for *CejaPI* which is expressed strongly in male ones and weakly in female ones, the remaining target genes are barely expressed in juvenile leaves.

For A-class genes, *cejaAP1* has similar expression patterns between male and female buds, expressed in inner scales, stipules and bracts. *CejaFUL* is expressed in all scales, stipules and bracts of male and female buds as well as in carpels. *CejaFUL-like* is almost only expressed in bracts. *CejaAGL6* shows different expression patterns between male and female flowers, with relatively strong expressions in the outer scales of males while weakly in those of females, but expressed relatively week in carpels and stipules. Elsewhere, *CejaAGL6* is detected in female bracts but not in male ones. B-class genes are expressed in almost all male floral organs, especially *CejaPI* which is barely expressed in female buds. *CejaAP3_1* is expressed most often in both male and female bracts. This *CejaAP3_1* is expressed most in both male and female bracts, where the expression level of *CejaAP3_1* is 3–4 times compared with *CejaActin*. *CejaAP3_2* is expressed higher than *CejaAP3_1* in stamens and carpels, but in both male and female bracts, expression level of *CejaAP3_2* is much less than *CejaAP3_1*. Apart from this observation, we found that, *CejaAP3_1* displays a similar expression pattern with *CejaAP3_2* between other male and female floral parts (low level). For C-class genes, *CejaAG1* is mainly expressed in carpels, stamens and both bracts. *CejaAG2* is expressed in carpels and both bracts (low level), but less than *CejaAG1*. The D-class gene *CejaAGL11* is expressed quite strongly in carpels.

## Discussion

Since species identification and classification are based on morphology, an increasing number of studies suggested that sole reliance on this approach may lead to the neglect of a significant number of relevant species [25]. As the development of molecular phylogenetics, DNA and amino acid sequence analyses have been an important method to study systematic evolution and development. As Woese [26] argues, sequencial information contains the promise that we will have potentially more evolutionary information than we now possess and allows us to infer a great deal of assurance than we can now.

### MADS-box homologs and systematic place

**Fig 8:**
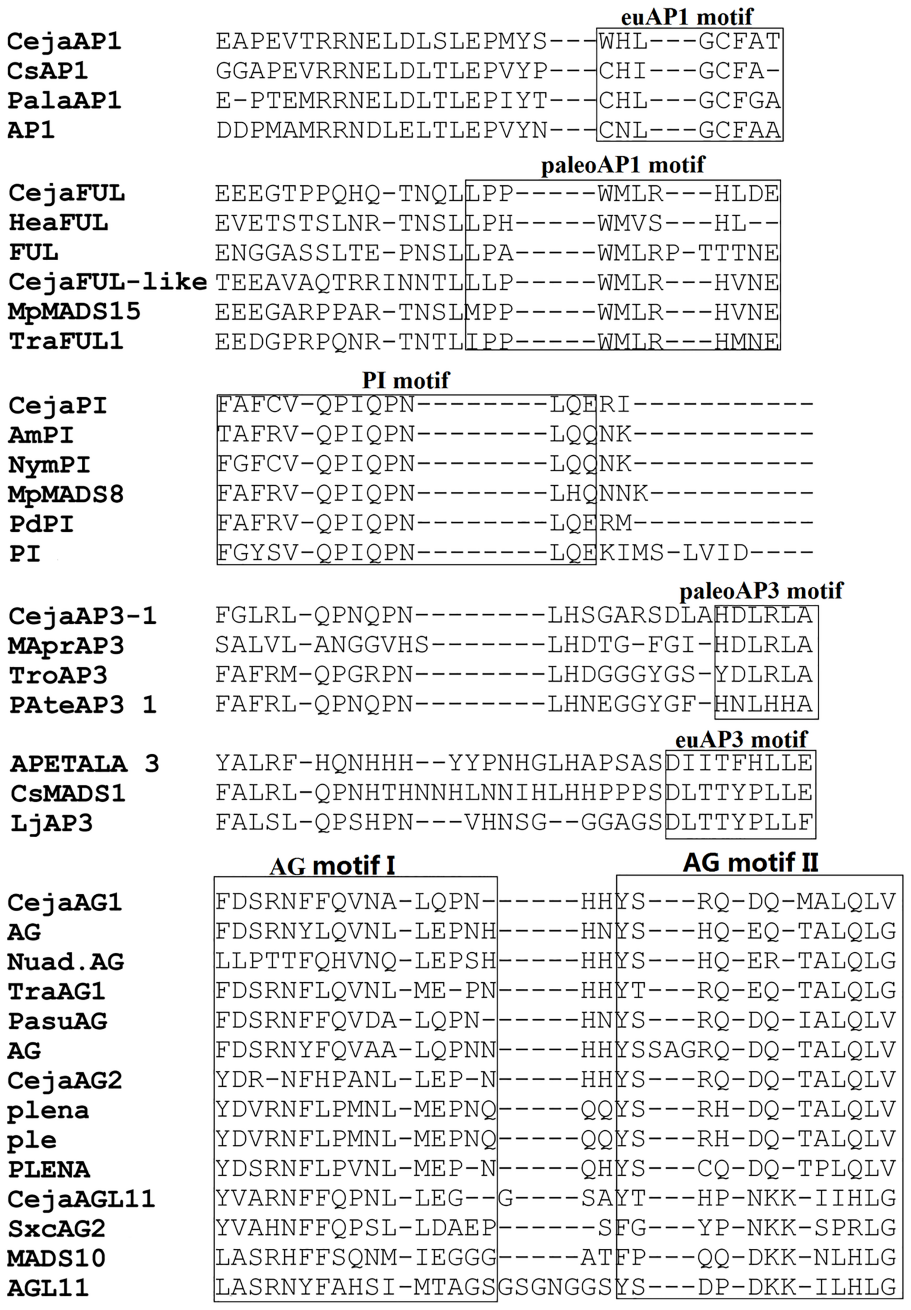
Representative predicted amino acid sequences of ABCD genes from *Cercidiphyllum japonicum* and selected taxa.

We obtained three A-class, three B-class, two C-class homologs and one D-class homolog from *Cercidiphyllum japonicum*, which has never been reported before. Phylogenetic analyses show that these floral organ identity genes group with the respective classes of the MADS-box genes from other *Saxibragales* plants, indicating that placing *Cercidiphyllum japonicum* in *Saxibragales* in the basal core eudicots is suitable. The C-terminal regions of *C*. *japonicum* genes contained conserved characteristic motifs, typical of the genes of each class (Fig 8), therefore indicating their functional similarities with other homologs regulating flower formations in other plants [27,28].

Only the C terminal is shown. Conserved motifs are boxed, as defined by previous studies for the AP1 motif, the PI and AP3 motifs, and the AG motif.

Recent studies suggested that the major duplication events for floral ABC-class genes occurred at the base of core eudicots [29–32]. For A-class genes, it has been proposed that a major duplication event occurred near the base of their core eudicots, giving rise to *euAP1*, *euFUL* and *FUL*-like lineages [31,33,34]. All the three A-class lineages we obtained from *C*. *japonicum*, thus suggesting that it could have originated after this duplication period. For the *AP3*/*PI* subfamily, one duplication formed *DEF*/*AP3* (*paleoAP3*) and *GLO*/*PI* lineages. Subsequently, following the duplication in the base of core eudicots, a frame shift mutation occurred in *DEF/AP3* copies and formed *TM6* and eu*AP3* lineages [29,35]. Predicted amino acid sequense of *CejaAP3_1* contains a *paleoAP3* motif, suggesting that *C*. *japonicum* may not originate may not have originated later than the base of the core eudicots. In addition, euAG- and PLE-lineage originated on account of a major duplication in the early period of core eudicots and undergone the functional switch between them after rosid and asterid differentiations [30,36,37]. Since both *euAG* and *PLE* homologs were found in *C*. *japonicum*, it is further demonstrated that *C*. *japonicum* may not have originated earlier than the rosid and asterid divergent period. Hence, the summation of molecular evidence limited the systematic place of *C*. *japonicum* to the base of core eudicots.

Studies of earlier ABCDE-models were based on the *Arabidopsis* and *Antirrhinum* model systems [16,17]. Based on ABCDE-model, we speculated that the sexual differentiation of C. japonicum may be related to the B-/C-class homologs. In the most recent common ancestor of gymnosperms and angiosperms, the primitive function of *AG* lineage was to differentiate the reproductive organs from nutritional organs [38,39]. The function of *DEF/GLO* lineage is to differentiate male and female [40]. The B-class gene *SlAP3Y* in *Silene latifolia* is located in the Y chromosome and related to gender decision [41]. The qRT-PCR results show that *CejaAG1* is highly expressed in stamens and carpels, while the *CejaAG2* is almost only expressed in carpels strongly. Previous studies have indicated that the B-class genes of core eudicots are stably expressed in petals and stamens, but this is not always coincident with the B-class genes of basal eudicots and basal angiosperms [42]. For instance, the *CejaPI* is almost male specific, since it is strongly expressed in all male organs and barely examined in female ones. These results may indicate that *CejaAG1* plays an important role in reproductive organ formation. As well, *CejaAG2* and *CejaPI* are crucial to carpels and stamens in floral development of *C*. *japonicum* respectively. Since functional verification is difficult to conduct in woody material, evidence for functions of identified ABCDE genes of *Cercidiphyllum japonicum* should use the corresponding mutant *Arabidopsis* as medium in future studies.

### Confusing structure of *C*. *japonicum*

In general, *C*. *japonicum* is thought to be missing the perianth. When we observed the male and female inflorescences, we encountered that there were two lamelliform and membranous bracts in male while there were four in female ones. Ding [43] described the ‘bracts’ as four sepals in the *Flora of Henan*. In another point of view, Yan et al. [15] observed morphogenesis of *C*. *japonicum* and considered that bracts should be closer to phyllome, but the so called bracts in *C*. *japonicum* developed with their basal stamens or pistils correlatively; hence they proposed that the so called bracts are more closely related to tepals. We found that leaf buds and flower buds are much the same except for their reproductive parts. According to the *Agricultural Dictionary*, a bract is actually a phyllome. Based on the model, the absence of petals in *C*. *japonicum* might be due to the null function of A-class and B-class homologs. The *APl/SQUA* family, such as *AP1* mutant of *Arabdopsis* and *SQUA* mutant of *Antirrhinum majus*, may cause changes of petals and sepals [33,44,45]. Moreover, the petals were converted to sepals and stamens to carpels in the *ap3* and *def* mutants [40]. Unfortunately, definite evidence of A-class homologs has never been demonstrated in woody plants and the expression patterns are not strictly conserved. In most primitive angiosperms, it is the petals not bracts or sepals having high expression levels of both A- and B-class genes, such as Orchid [46,47], *Trochodendron* [48] and *Eucalyptus* of *Saxifragales* [49]. Wróblewska et al. [50] analyzed expression patterns of key flower genes of several *Magnoliaceae* and found that the B-class genes, *AP3* and *PI*, were restricted to the second and third whorl. In our research, the qRT-PCR results show that both A- and B-class genes, especially *CejaAP1* and *CejaAP3_1/_2* whose homologous genes are petal decisive in *Arabidopsis*, had significant expressions in the bracts that are different from other organs of *C*. *japonicum*. Recent studies in *Arabidopsis* and *Antirrhinum*, as well as several other species, indicate that the function of floral MADS-box genes is largely associated with the expression patterns of these genes, particularly when expression levels are high [51]. What is more, the epidermal cells of the bracts show considerable differences from other phyllomes. In view of this inference, we recommended that the so-called bracts actually should be considered as perianth.

### Exon skipping of *CejaAP3*

**Fig 9:**
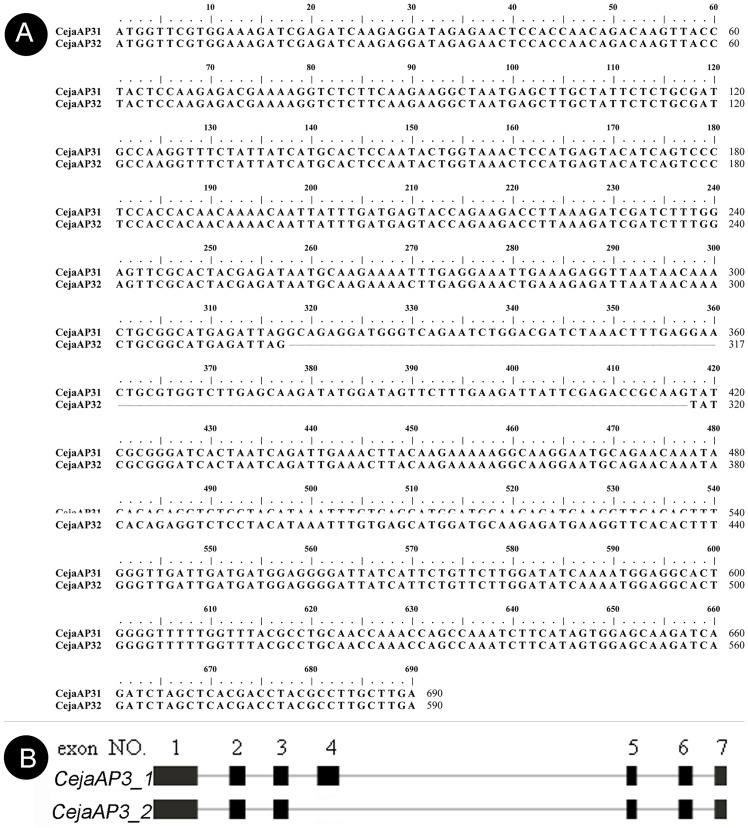
The alignment of the two *CejaAP3* transcripts. (A) showing sequence alignment. (B) showing alternative splicing.

Alternative splicing has been found in several MADS-box genes which, to some extent, might have either an important positive or negative impact, typical in Magnolia stellata [52]. During the screening, two CejaAP3*_1/_2* spliceosomes were found. After examining the genomic sequence, we found that the two clones may be formed by alternative splicing. In addition, the shorter spliceosome, *CejaAP3_2*, was confirmed to be missing an exon 4 (Fig 9). What is more, the results of qRT-PCR shows that *CejaAP3_2* displays a high expression in stamens and moderate expression in other floral parts, indicating that this abnormal splicing may have a significant impact on the floral development of *C*. *japonicum*, especially the perianth. However, the exact nature of this product and its interactions need further study.

We conclude that all floral homeotic gene phylogenies show that *C*. *japonicum* is closely related to the plants of *Saxifragales*, suggesting that our species should be placed in *Saxifragales* at the base of core eudicots. This result confirms the APGIII system and supports a new train of thought when investigating systematic evolution based on floral organ identity genes. As well, our research supports the conjecture that the so called bracts of *C*. *japonicum* actually are perianth, a conclusion based on morphology and expression patterns.
